# Closing the sepsis gap: from molecular mechanisms to scalable bedside care

**DOI:** 10.1038/s41598-026-63958-2

**Published:** 2026-08-05

**Authors:** Luis Felipe Reyes, Krzysztof Laudanski

**Affiliations:** 1 Unisabana Center for Translational Science, Universidad de La Sabana, Chía, Colombia; 2 Clínica Universidad de La Sabana, Chía, Colombia; 3 ISARIC, Pandemic Sciences Institute, University of Oxford, Oxford, UK; 4 Department of Anaesthesiology and Perioperative Care, Mayo Clinic, Rochester, MN USA

**Keywords:** Diseases, Microbiology

## Abstract

Recent estimates point to roughly 49 million cases and 11–13 million deaths annually due to sepsis, with a disproportionate burden borne by low- and middle-income countries. Three decades of progress, summarised in the recently updated Surviving Sepsis Campaign guidelines, have refined how clinicians screen for, resuscitate, and treat patients with sepsis. Yet, persistent uncertainty surrounds early identification, pathogen detection, hemodynamic targets, adjunctive therapies, and the long-term burden carried by survivors. This Collection, Sepsis: Treatment, intervention, mortality, brings together original research that maps where the field is moving: pragmatic diagnostics that run on standard hospital equipment; multi-omic biomarker discovery in blood, plasma extracellular vesicles, and urine; computational dissection of pathogen biology and longitudinal host-response trajectories; preclinical interrogation of metabolic and signalling pathways implicated in organ injury; and mechanistic studies of the muscle and mitochondrial wasting that shape life after sepsis. Together, these papers sketch a research agenda for sepsis that is simultaneously molecularly precise and operationally scalable.

## Sepsis research and care at an inflexion point

After three decades of guideline-driven improvement, sepsis still kills more people each year than ischaemic stroke, road traffic injury, or breast cancer. The most recent Global Burden of Disease estimates put the annual toll at roughly 11 million deaths, with around 85% occurring in low- and middle-income countries (LMICs).^1^ The 2026 Surviving Sepsis Campaign (SSC) guidelines reflect both how far we have come and how challenging the remaining gaps are. The panel issued recommendations on screening, infection management, hemodynamics, ventilation, adjunctive therapies, and post-acute care, but acknowledged that much of the underlying evidence remains low- or moderate-certainty and that many recommendations are conditional and resource-sensitive.^2^ For frontline clinicians, the question is no longer whether to deliver evidence-based sepsis care, but how to deliver it reliably with the resources at hand.

This Collection, gathered under the theme of Sepsis: Treatment, intervention, mortality, was conceived as a venue for original research to help close this gap. The accepted manuscripts cluster around three priorities that mirror the most pressing questions now being asked of sepsis evidence reviews worldwide: how can we identify and characterise sepsis earlier and more accurately, how can we modulate the host response to improve outcomes, and how can we mitigate the long-lasting morbidity?

## Earlier and more accurate diagnosis

Time to diagnosis remains the bottleneck of sepsis care, and several papers in this Collection address it from complementary angles. Marino Miguélez and colleagues describe a centrifugation-based protocol that recovers up to 85% of bacteria from inoculated blood culture media within minutes and yields species identification in under twelve hours using standard laboratory equipment and chromogenic agar, an approach with obvious appeal in settings where MALDI-TOF or molecular platforms are unavailable.^3^ Nacke and co-workers similarly demonstrate that the Intensive Care Infection Score, derived from routine cellular haematology parameters, remains stable in K_3_-EDTA whole blood for at least eight hours at room temperature, supporting its use in centres where sample processing may be delayed.^4^ These pragmatic studies sit alongside molecular and computational work that points to the next generation of diagnostics. Park and colleagues identify hundreds of differentially expressed proteins in plasma extracellular vesicles, mapping signatures of innate immunity, coagulation, and endothelial activation that correlate with sepsis endotypes and mortality.^5^ Moreover, Zeng and colleagues develop a urinary proteomic nomogram (SLC25A24, UBQLN1, CREB3L3) for non-invasive prognostication;^6^ Nunnari and co-workers use a machine-learning framework to show that low plasma glutamic acid predicts myelosuppression and 30-day mortality;^7^ and Thon and colleagues extend the biomarker conversation to viral sepsis, linking elevated soluble Delta-like canonical Notch ligand 1 to secondary infection, organ dysfunction, and 90-day mortality in COVID-19.^8^ Chen and colleagues bring artificial intelligence to the pathogen side, applying contextualised DNA language models to characterise the regulatory grammar of sepsis-causing *Staphylococcus aureus* strains, an approach with direct implications for understanding antimicrobial resistance.^9^

## Sepsis pathology and therapy

If the first task of sepsis research is to know who is septic, the second is to know what to do. Several papers in this Collection illustrate where mechanism-driven therapeutic discovery is heading. Kim and colleagues revisit vitamin C, a much-studied and largely disappointing adjunct in clinical trials, and ask whether prior negative results reflect a regimen that was “too late, too little, and too short”; in a murine cecal ligation and puncture model, early, very high-dose, and prolonged ascorbic acid attenuated lung, kidney, and liver injury and improved survival.^10^ An *et al.* map the longitudinal whole-blood transcriptome of 128 hospitalised COVID-19 patients across six disease phases, identifying dynamic shifts in adaptive immunity, haemostasis, and metabolism, and using drug-gene set enrichment to flag candidate repurposed therapies.^11^ Together with the pathogen-side work above, these manuscripts illustrate a maturing systems view of sepsis in which patient heterogeneity is not a nuisance to be averaged out, but a target to be understood.

## Surviving sepsis, beyond the hospital

A particularly welcome strand in this Collection concerns what happens after the acute illness. Romien and colleagues show in a murine model that sepsis induces persistent skeletal muscle weakness and mitochondrial dysfunction three months after recovery, with low-grade inflammation, NLRP3 inflammasome activation, and engagement of the receptor for advanced glycation end-products (RAGE) axis; in RAGE-knockout animals, these long-term sequelae do not occur.^12^ Ono and colleagues converge on a related target, demonstrating that pharmacological inhibition of STAT3 signalling with C188-9 attenuates sepsis-associated muscle wasting in vivo and in vitro, with parallel signals in human patients.^13^ Sepsis survivorship has been one of the field’s blind spots; these mechanistic studies make an explicit case for treating long-term morbidity as a primary outcome rather than an afterthought.

## From the bench to a globally relevant bedside

The manuscripts in this Collection do not, on their own, change clinical practice, but together they sketch the contours of where sepsis research must go to serve patients worldwide. Three lessons stand out. First, equity is a methodological choice: diagnostic innovations that depend on infrastructure absent from most of the world’s hospitals will not narrow the global mortality gap, and contributions such as the rapid blood-culture protocol and the time-stable haematology score are reminders that pragmatic science is precision science when the patient is in a district hospital. Second, multi-omic and machine-learning approaches explore hypotheses faster than clinical trials can test them; this heightens the urgency of building international, biobanked cohorts that include patients from LMICs, so that the biomarkers and endotypes we validate are the ones our patients need. Equally important is the need to reconnect mechanistic discovery with pragmatic clinical investigations. Many of the pathways highlighted in this Collection, including endothelial injury, immune dysregulation, and metabolic dysfunction, are biologically plausible but clinically unvalidated targets. Bridging this gap will require adaptive, internationally coordinated research platforms that link deep phenotyping to embedded interventional studies. Such infrastructure is particularly important in LMICs, where the epidemiology, pathogen landscape, antimicrobial resistance patterns, and resource constraints of sepsis differ substantially from those of high-income settings that dominate most biomarker and therapeutic literature. Without more inclusive geographic representation, there is a real risk that precision medicine approaches in sepsis will become precise only for the populations already overrepresented in research. Third, the agenda set by the SSC and contemporary evidence-review programmes, around early identification scores, microbiological diagnostics, fluid and vasopressor strategy, antimicrobial timing, source control, corticosteroids, and organisational care pathways, aligns naturally with the themes of this Collection.

Sepsis is a heterogeneous syndrome in which the underlying mechanisms of disease differ; treatment decision-making should be driven by this to provide personalised guidelines. The patients we serve do not have the luxury of waiting another decade. The challenge for the field is to keep our science ambitious and our delivery feasible, in equal measure.
